# Protein domain movement involved in binding of belinostat and HPOB as inhibitors of histone deacetylase 6 (HDAC6): a hybrid automated-interactive docking study

**DOI:** 10.1007/s10822-025-00636-x

**Published:** 2025-07-15

**Authors:** Georgios Iakovou, L. Palmer, A. Ganesan, Akio Kitao, Stephen D. Laycock, Steven Hayward

**Affiliations:** 1 Digital Engineering, Aviva Plc, Norwich, Norfolk, NR1 3NS UK; 2 School of Computing Sciences, University East Anglia, Norwich, NR4 7TJ UK; 3 School of Chemistry, Pharmacy, and Pharmacology, University East Anglia, Norwich, NR4 7TJ UK; 4 School of Life Science and Technology, Institute of Science Tokyo, Tokyo, 152-8550 Japan

**Keywords:** Drug-design, Molecular-recognition, Linear-response, Receptor flexibility

## Abstract

DockIT is a tool for interactive molecular docking that can model both the local and global conformational response of the receptor to the docking of a ligand based on information from a molecular dynamics simulation. Using DockIT we have investigated the binding process of two histone deacetylase (HDAC) inhibitors to HDAC6: the nonselective approved drug belinostat and the preclinical HPOB. To model HDAC6’s conformational response to the binding of the inhibitors we performed a 200-nanosecond explicit-solvent molecular dynamics simulation on HDAC6. Unexpectedly the simulation revealed a domain movement that affects the size and shape of the binding pocket. Using automated docking and a rigid model for the inhibitors, the domain movement continuously adapts the pocket to the presence of the inhibitor. For both inhibitors, an intermediate binding site was found where it was partially inserted, with a hydrogen bond formed between the inhibitor’s hydroxamic acid and the Tyr745 side chain. Pushing the inhibitor deeper into the pocket over an energy barrier and re-engaging automated docking, a final binding pose resulted with a root-mean square deviation with its respective crystallographic pose of 1.0 Å for belinostat and 1.4 Å for HPOB. We believe our results mimic substrate recognition by the enzyme, with an initial partial binding of the acetyllysine residue with Tyr745. During binding a relay of hydrogen bonds occurs coordinating the orientation of the cap and the hydroxamic acid inside the pocket. The interaction between the cap and the surface of HDAC6 explains the reason for the hydroxamic acid warhead in HPOB binding in a flipped orientation compared to belinostat.

**Supplementary Information:**

The online version contains supplementary material available at 10.1007/s10822-025-00636-x.

## Introduction

**Fig. 1:**
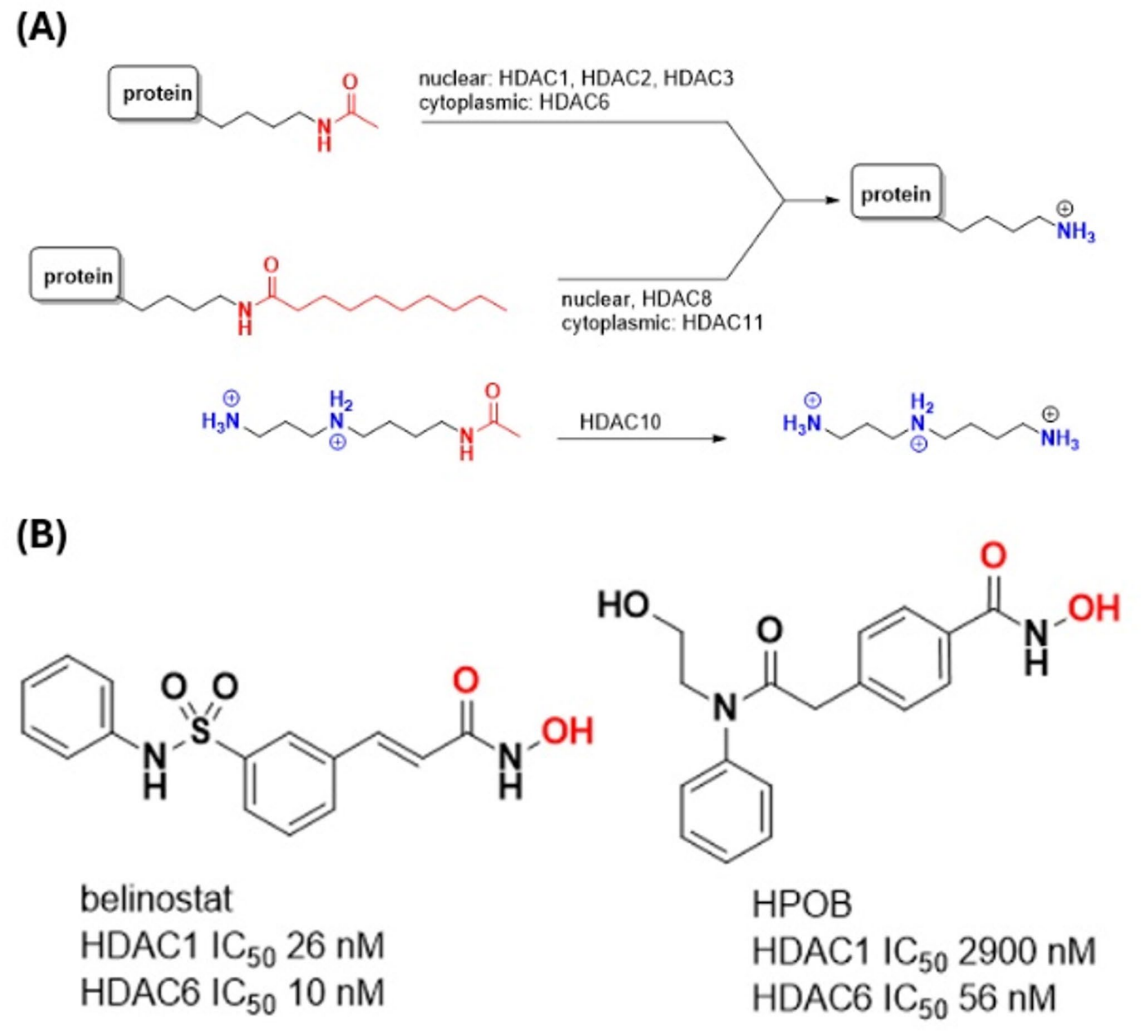
(**A**) The substrate scope of human histone deacetylases (HDACs): removal of acetyllysine and fatty acid acyllysine PTMs in proteins, or the deacetylation of polyamines (**B**) The HDAC inhibitors investigated in this study, with the hydroxamic acid highlighted in red. Reported potencies against HDAC1 and HDAC6 are given as a measure of inhibitor selectivity

In eukaryotic organisms, epigenetics refers to the reversible modifications of DNA, nuclear proteins and RNA that are coordinated to modulate gene expression. In epigenetics, the acetylation of lysine residues in histone tails plays a central role. This protein post-translational modification (PTM) is introduced by histone acetyltransferases (HATs) and converted back to the native lysine residue by the action of histone deacetylases (HDACs). Eukaryotic species have evolved to produce multiple HDACs that perform distinct physiological functions (see Fig. 1(A)). Humans have eleven HDACs with HDAC1, HDAC2 and HDAC3 playing the classical role of acetyllysine protein hydrolysis in the nucleus. Histones are a major substrate and the action of these HDACs serves to repress gene transcription by opening up chromatin. HDAC6 performs a similar deacetylating function in the cytoplasm, acting on substrates like tubulin and cortactin. Two additional members of the family, HDAC8 and HDAC11, are more efficient at hydrolysing acyllysine residues with long-chain fatty acylamides than acetyllysine. HDAC10 is an outlier, as its substrate is the polyamine acetylspermidine rather than a protein, while HDAC4, HDAC5, HDAC7 and HDAC9 are pseudoenzymes that are catalytically incompetent.

**Fig. 2:**
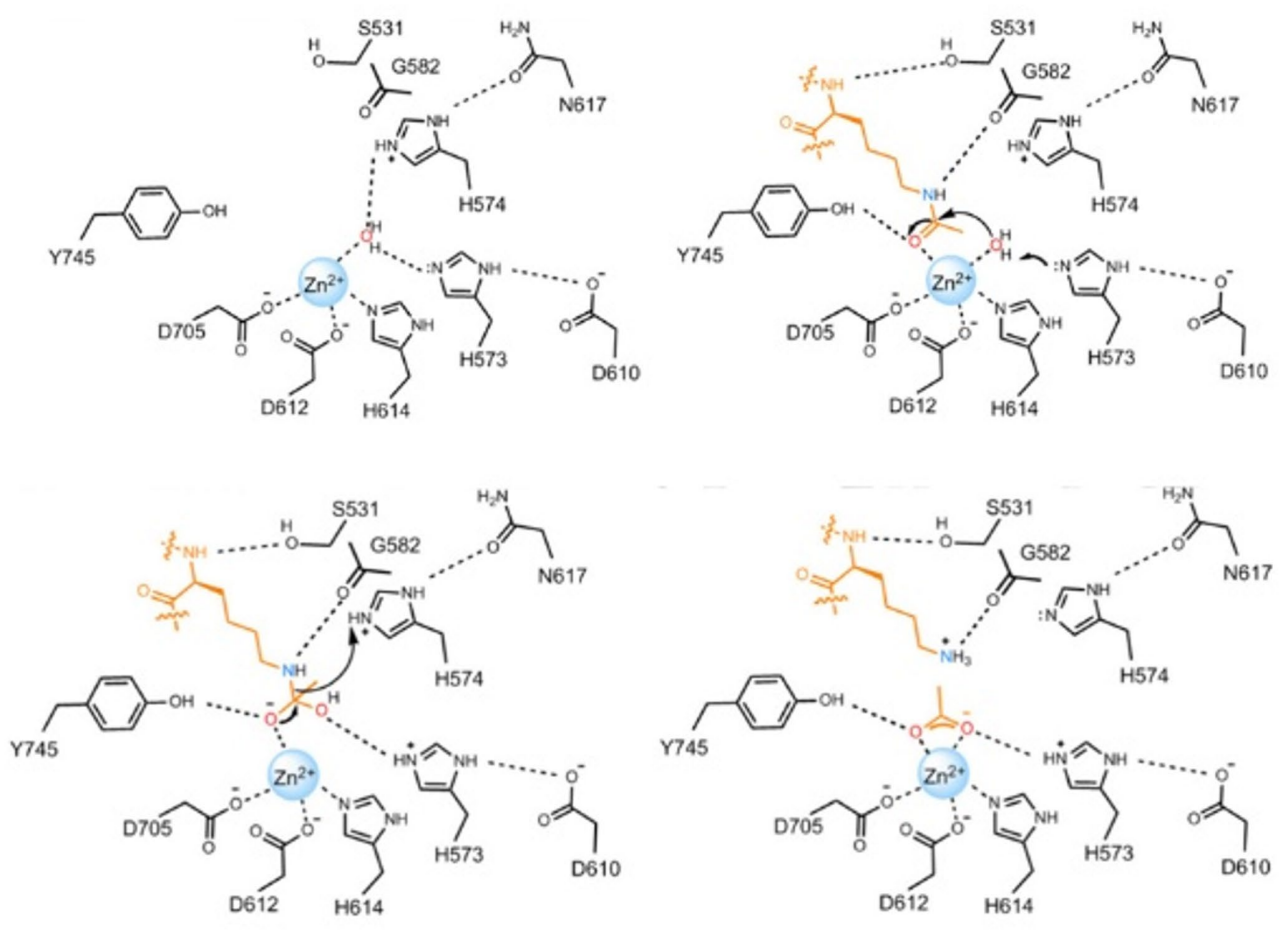
The proposed catalytic mechanism of HDAC6. Adapted from reference [1] with permission. Copyright 2024 American Chemical Society

The HDACs contain an active site zinc cation that coordinates to the carbonyl group of the acetyllysine PTM as well as the incoming water nucleophile, thereby reducing the activation energy for hydrolysis of the amide bond. Extensive X-ray crystallographic studies have produced a unified hypothesis of the HDAC catalytic mechanism: the acetyllysine PTM is activated through noncovalent interactions with the zinc cation and a conserved gatekeeper Tyr residue (see Fig. 2) [1, 2].

Due to the dysregulation of HDAC expression or activity in human disease, their inhibition has become the most important of the epigenetic targets for drug discovery [3–5]. Tens of small molecule inhibitors that achieve potency through reversible coordination to the zinc cation have advanced into clinical development, and five are Food and Drug Administration (FDA) approved. The latter share common structural motifs: a zinc binding ‘warhead’ attached by a linker to a ‘cap’ (the end of the molecule opposite to the hydroxamic acid). In the natural product prodrug romidepsin, a thiol is the ‘warhead’, while the others - vorinostat, belinostat, panobinostat and givinostat - are synthetic compounds with hydroxamic acid ‘warheads’. The hydroxamic acid mimics the interactions in the tetrahedral transition state through bidentate coordination of the zinc, as observed in protein-inhibitor cocrystal structures.

The clinically approved hydroxamic acids are relatively indiscriminate between the eleven human HDACs, leading to tolerability issues that limit their utility to a narrow spectrum of hematological tumors. More recent efforts have targeted HDAC6 with the view that selective inhibition of this cytoplasmic enzyme would reduce side effects compared to the global disruption of transcription that occurs when interfering with nuclear histone deacetylases [6, 7].

Breslow, who discovered vorinostat, the first FDA approved HDAC inhibitor, reported the lead *N*-hydroxy-4-(2-[(2-hydroxyethyl)(phenyl)amino]-2-oxoethyl)benzamide (HPOB) as an early example of an HDAC6-selective compound [8].

In this manuscript we investigate the interactions of two inhibitors, the selective HPOB and non-selective belinostat (see Fig. 1 (B)), with HDAC6 using a flexible protein docking approach [8, 9].

**Fig. 3:**
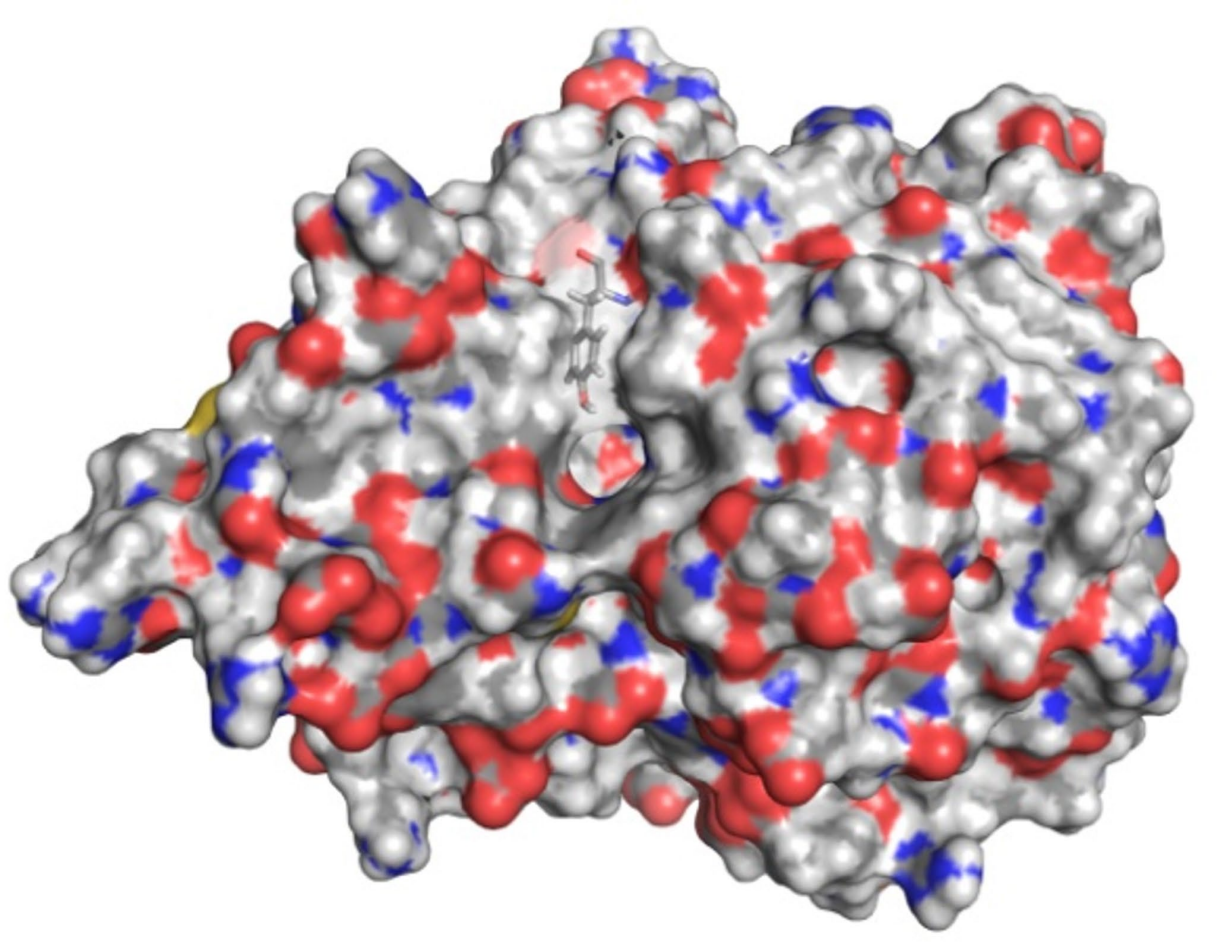
View of HDAC6 receptor structure used for docking, with binding pocket facing towards the viewer in the center of the structure. Directly above the pocket is Tyr745 depicted in stick model. This receptor structure is the CD2 domain derived from the unliganded *Danio rerio* HDAC6 structure, PDB: 5EEM. See main text for details

HDAC6 comprises a tandem repeat of catalytic domains, denoted CD1 and CD2, which differ in their specificity [10]. The structures used here are from the CD2 domain. Figure 3 shows a view of the HDAC6 structure indicating the binding pocket and the conserved gatekeeper residue, Tyr745.

Several MD simulations of HDAC6 have been reported in the literature [11–17]. All these investigations performed MD simulations on HDAC6 in complex with an inhibitor that was computationally docked using automated methods. The papers report on residue-ligand interactions and the stability of the complex by monitoring root mean-square deviation (RMSD) trajectories and/or root mean-square fluctuations (RMSFs) of individual residues. Shahab et al. [17] presented principal component analysis (PCA) projections of 100 ns MD trajectories of HDAC6 in complex with various inhibitors, each presenting a different distribution in the projected space. None of these publications reported on the mechanism by which the initial protein-ligand recognition occurs or the kind of protein conformational changes that guide access to the enzyme active site.

Although not directly related to this work, MD simulations have been performed on other proteins that bind to acetyllysines in histones, such as bromodomains [18].

In contrast to automated docking tools, interactive docking tools, some of which are implemented in VR [19], are comparatively few in number. Examples include DockIT [20], DockPro [21] IMD [22] and UDock2 [23]. Interactive tools are not suited for predicting binding sites due to the large search space but can be used in structure-based drug design (SBDD) where a multi-participant immersive environment can foster a collaborative approach to lead optimization. They are also ideal for educating students about biomolecular function [24]. In interactive docking the relative position and orientation of the ligand and receptor is controlled by the user and interaction forces between the two molecules are calculated. For DockIT, methods were developed to calculate interaction forces in real time [25–27] so that it could include molecular flexibility. For flexible receptor docking, the method of linear response is used to calculate the conformational response of the receptor to forces from the ligand. Flexible receptor docking was proven to be successful in the Haptimol FlexiDock prototype [28] and receptor flexibility was then incorporated into DockIT together with a virtual reality (VR) interface using a headset and hand-held controllers [29].

Here we use DockIT to investigate a promising protein target and its interaction with potential drug molecules. Specifically, this study has two main aims: the first is to see if DockIT can be used to predict binding poses for two inhibitors, belinostat and HPOB; the second is to determine what kind of conformational response occurs in HDAC6 during the binding process.

## Methods

### DockIT

The methods underpinning DockIT have been described in previous publications [20, 28, 29]. Within the framework of linear response, DockIT uses a gradient descent procedure to bring the receptor smoothly into static equilibrium [28], where the vector of atomic coordinate displacements of the receptor atoms due to forces of interaction with the ligand, $\:\varvec{\Delta\:}\varvec{r}$ (a 3*N* ×1 matrix, where *N* is the number of receptor atoms), satisfies the following “static equilibrium” equation:

1 $$\:\varvec{\varDelta\:}\varvec{r}=\frac{1}{{k}_{B}T}{\varvec{V}}_{M}{\varvec{\lambda\:}}_{M}{\varvec{V}}_{M}^{t}\varvec{f}\left({\varvec{r}}_{\text{o}}+\varvec{\varDelta\:}\varvec{r}\right)$$.

Where $\:{k}_{B}$ is Boltzmann’s constant, $\:T$ the absolute temperature, $\:{\varvec{r}}_{\text{o}}$ (3*N*×1) the vector of coordinates for the relaxed receptor structure, and $\:\varvec{f}$ (3*N*×1) the vector of forces on receptor atoms from the ligand. The matrices $\:{\varvec{\lambda\:}}_{M}$ (*M*×*M* diagonal) and $\:{\varvec{V}}_{M}$ (3*N*×*M*) ($\:{\varvec{V}}_{M}^{t}$ denotes the transpose of $\:{\varvec{V}}_{M}$) contain the first *M* eigenvalues and eigenvectors of the variance-covariance matrix of atomic displacements, respectively, derived from the trajectory of the MD simulation of the receptor [28]. *M* and *N* determine the number of multiplications required to evaluate $\:\varvec{\Delta\:}\varvec{r}$ in Eq. (1); *M* being chosen so that Eq. (1) can be evaluated in real time and depends therefore on speed and memory of the GPU being used [28]. Details of the MD simulation are given in the “MD simulation” section and details on the calculation of $\:{\varvec{r}}_{\text{o}}$, $\:{\varvec{\lambda\:}}_{M}$ and $\:{\varvec{V}}_{M}$ are given in the “PCA” section.

#### Total energy including strain energy

The linear response model used in DockIT effectively determines the response of the receptor to external forces from the ligand using the elastic approximation, the parameters of which are derived from the trajectory of an MD simulation. Any distortion of the receptor from its relaxed conformation will result in strain and the strain energy can be evaluated. Using *M* eigenvalues and eigenvectors, the strain energy is given by:

2 $$\:{E}_{strain}=\frac{1}{2}{k}_{B}T{\varvec{\Delta\:}\varvec{r}}^{t}{\varvec{V}}_{M}{\varvec{\lambda\:}}_{M}^{-1}{\varvec{V}}_{M}^{t}\varvec{\Delta\:}\varvec{r}$$.

The strain energy is added to the interaction energy for display as the total energy in the energy trajectory plot window.

#### Automated docking

We have implemented an automated docking method that uses a gradient descent procedure. As reported previously we use gradient descent to move smoothly towards the receptor conformation that is in static equilibrium [28, 29]. This is applied to the receptor coordinates only, the ligand position and orientation being under the control of the user. By pressing the “Auto” button, steepest descent for the rigid ligand can be performed whereby the ligand is translated and rotated according to the total force and torque acting on it from the receptor. Before each step of steepest descent for the ligand, the receptor is brought into static equilibrium, i.e., it satisfies Eq. (1). This is often referred to as a quasi-static process.

In this study we increase the cut-off distance for the non-bonded interactions from its default 8 Å to 100 Å. Using this long cut-off distance means that no sudden jumps in interaction energy occur as the cut-off boundary is crossed during gradient descent.

#### Ghost ligand

A ghost ligand can be seen but does not interact with either the ligand or the receptor and cannot be moved independently of the receptor. In this study, the inhibitor molecule in its experimentally bound pose with HDAC6 is a ghost ligand to indicate where the ligand inhibitor would be expected to bind.

### Receptor and ligand selection

The zebrafish *Danio rerio* HDAC6 is widely used for X-ray studies as it is more reliable than the human enzyme in giving diffraction-quality crystals while maintaining a high sequence homology within the active site. The structure selected for the MD simulation was the ligand-free CD2 domain from zebrafish: PDB file: 5EEM (chain A) [10]. A ligand-free structure was selected as the theory of linear response states that the equilibrium fluctuations of the unperturbed system can be used to model the response to a perturbation, which in this case will arise from forces of interaction with the inhibitors that bind to HDAC6. This structure was missing residues His771 and Leu772 and the side chain of Asp770. These were modelled using a fitting procedure using the HDAC6 structure, PDB: 7O2R [30]. The N-terminal residues 435–441 are not resolved in any of the HDAC6 structures and are not included.

The structures of the HDAC inhibitors belinostat and HPOB were taken from PDB: 5EEN [10], and from PDB: 5EF7 [10], respectively. Both ligands were bound in the active site cavity, coordinating the zinc ion through the hydroxamic acid warhead. It is significant for this study to appreciate that HPOB binds with its hydroxamic acid group in a “flipped” (the angle of rotation is 140° rather than the 180° for a perfect flip) orientation to that found in belinostat.

### Preparation of files used in DockIT

To model a flexible receptor DockIT requires a structure file (containing the atomic coordinates of the receptor, $\:{\varvec{r}}_{\text{o}}$), a topology file (in Gromacs format), containing the information on the non-bonded atomic interaction parameters, and eigenvalue ($\:{\varvec{\lambda\:}}_{M}$) and eigenvector ($\:{\varvec{V}}_{M}$) files from the all-atom PCA. As the simulations were performed using Amber, the Amber topology file (“prmtop” file) used for the simulation was converted to a Gromacs topology file (“top” file) using the PARMED tool (https://parmed.github.io/ParmEd/html/index.html).      

To construct force field parameters for the ligands, the second generation of general Amber force field (GAFF2) was employed, and Austin Model 1-bond charge corrections (AM1-BCC) were used to determine partial changes [31] using the Antechamber tool in AmberTools23 [32]. Again, PARMED was used to convert the Amber “prmtop” files for the ligands to Gromacs “top” files.

### MD simulation

The simulation system of HDAC6 in solution was constructed by AmberTools23 [32] and the molecular dynamics (MD) simulation was conducted using the pmemd.CUDA module [33–35] in Amber22 [32]. HDAC6 was initially solvated in a cubic box of size 92.1 × 92.1 × 92.1 Å^3^ filled with 19,914 OPC water molecules [36] and 0.14 M KCl ions. The Amber parm19SB force field [37] was used for the protein. The 12-6-4 model for van der Waals interactions [38] was employed for the zinc ion so that the coordination around the zinc ion is properly reproduced. After energy minimization, 50 ns equilibration MD was conducted with positional restraints. After initialising with random velocities generated to reproduce the Maxwell distribution at 300 K, positional restraints were imposed on all heavy atoms included in the original PDB file (protein heavy atoms, the zinc ion and two potassium atoms) with a force constant of 1.0 kcal/mol/Å^2^. The subsequent 20 ns MD simulation was conducted with positional restraints imposed on protein main chain atoms, the zinc ion and the two potassium atoms with a force constant of 0.1 kcal/mol/Å^2^. A production run was then carried out for 200 ns without positional constraints imposed. During the entire MD simulation, the zinc ion was stably coordinated by three O_δ_ of two ASPs, N_δ_ of HIS, and two O of two water molecules. For interactive docking in DockIT, the Amber03 force field was used for the non-bonded interactions and the standard 12 − 6 model for van der Waals interactions was used for the zinc ion.

### PCA

The matrices $\:{\varvec{\lambda\:}}_{M}$ and $\:{\varvec{V}}_{M}$ in Eq. (1) for modelling the conformational response of the receptor to the binding of the drug molecules are eigenvalues and eigenvectors of the variance-covariance matrix of atomic displacements derived from the MD trajectory. Determination of these is a PCA of the atomic displacements which has been used extensively in application to protein dynamics. We refer to two recent reviews for those interested in details [39, 40]. The basic procedure involves removing atomic displacements between conformational snapshots due to an overall translation and rotation of the molecule by performing all-atom, mass-weighted least-squares best fits to a reference structure; evaluation of the average structure; the building of a variance-covariance matrix of atomic displacements from the average; and the diagonalization of this matrix to get a set of eigenvalues and eigenvectors. The eigenvalues and eigenvectors, sorted in descending order of the eigenvalues, are saved in two separate files for use in DockIT. The structure file used when modelling a flexible receptor using the theory of linear response should ideally be the average structure. However, the average is not a viable bonded structure. Therefore, we use the closest-to-average structure from the fitted trajectory as our receptor in DockIT– these are the coordinates in $\:{\varvec{r}}_{\text{o}}$ in Eq. (1).          

### Ghost ligand placement

Ghost ligands are placed in the binding site of the receptor molecule. To find the appropriate coordinates for a ghost ligand, the protein molecule of the crystallographic ligand-bound structure is superposed on the receptor molecule. The translation and rotation used for superposition are then applied to the ligand.

## Results

Comparison of the experimental structures shows that there is little difference between the ligand-bound structures and the ligand-free structure, suggesting that HDAC6 is a rather rigid structure (see Table I).

**Table I:** RMSDs between ligand-free structure (5EEM, chain A) and ligand-bound structures after least-squares superposition of C_α_ atoms

Ligand-bound: PDB (chain id, ligand name, PDB ligand code)	RMSD (Å)
5EEN (B, belinostat, 5OG)	0.17
5EF7 (A, HPOB, 5OJ)	0.14

### PCA on ligand-free equilibrium trajectory of HDAC6

**Fig. 4:**
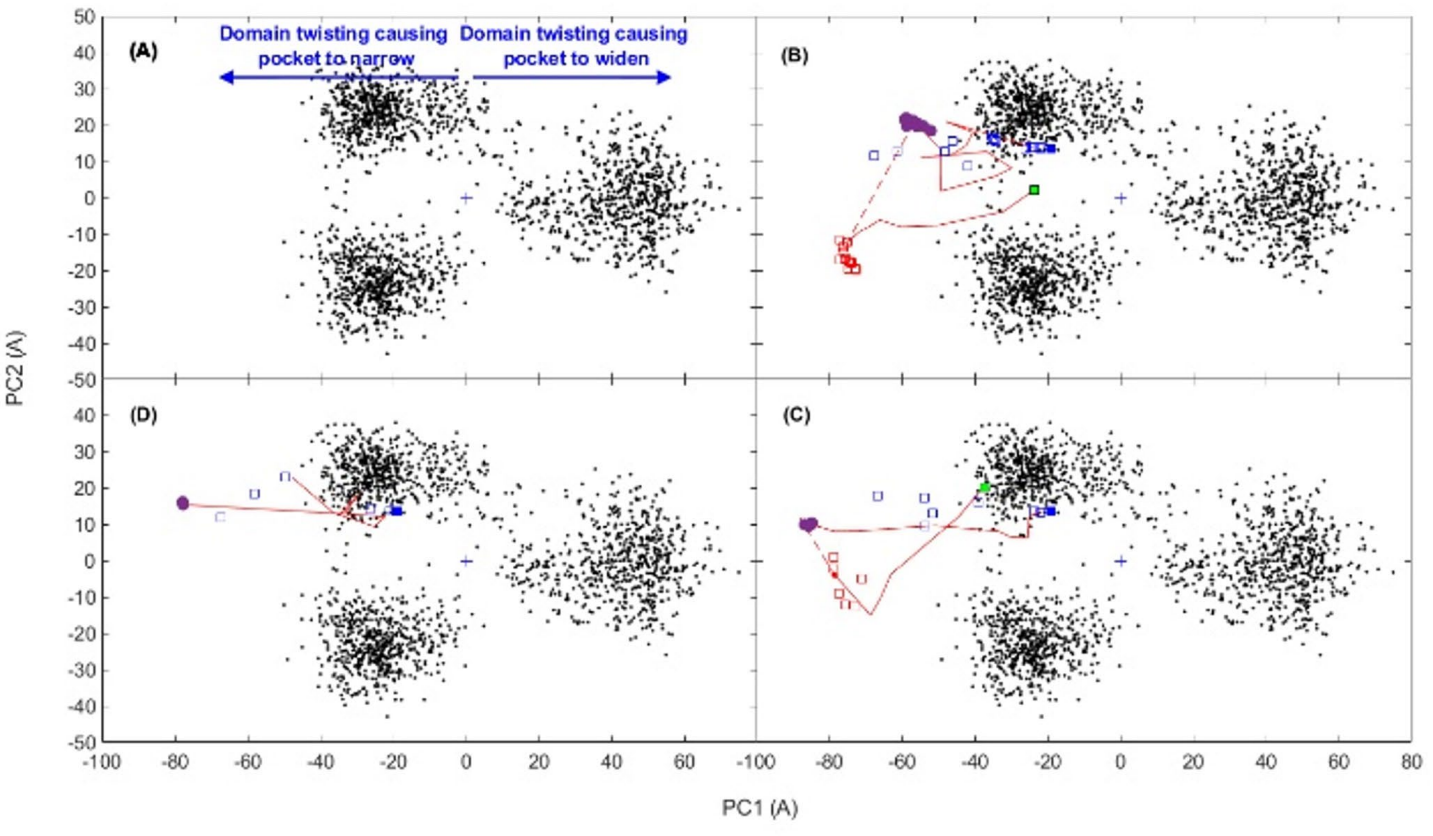
The black points are the 1800 frames from 20.1 ns-200 ns portion of trajectory projected on to the first two principal modes, PC1 and PC2. The red line shows a typical docking trajectory. Automated docking is indicated by a continuous line, and manual docking by a broken line. The blue filled square is the relaxed receptor conformation. The open blue squares correspond to the different starting poses. Purple circles are at the intermediate binding site where the side chain hydroxyl group of Tyr745 forms a hydrogen bond with the hydroxamic acid group. Open red squares are immediately after the push over energy barrier and green squares (all coinciding) indicate the final docked conformation (**A**) Movement in the direction of negative values for PC1 cause the binding pocket to narrow. Conversely movement in the direction of positive values for PC1 causes the binding pocket to widen (**B**) Docking of belinostat for the 14 cases (**C**) Docking of HPOB: the 7 cases where the hydroxamic acid group is oriented as in the HPOB-bound crystallographic structure (**D**) Docking of HPOB: the 5 cases where the hydroxamic acid group is in the same orientation as in belinostat

**Fig. 5:**
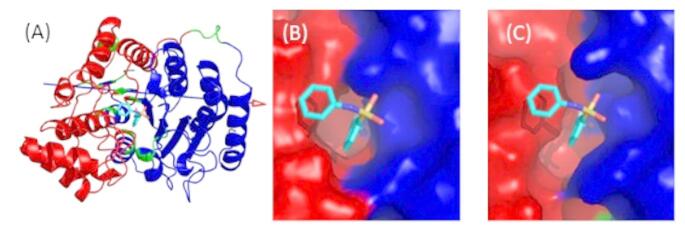
DynDom result where input is the two extreme projections $\:{\varvec{r}}_{1}^{min}$ and $\:{\varvec{r}}_{1}^{max}$ on the first principal mode from the PCA of the HDAC6 MD trajectory. The dynamic domains are in red and blue, hinge bending regions in green. In stick depiction is the belinostat ligand fitted into the active site. The domain boundary bisects the binding pocket indicating that the domain motion affects its size and shape. (**A**) Cartoon depiction showing hinge axis as an arrow (**B**) At the minimum value, $\:{\varvec{r}}_{1}^{min}$ (**C**) At the maximum value structure, $\:{\varvec{r}}_{1}^{max}$, where the binding pocket has widened in comparison to the $\:{\varvec{r}}_{1}^{min}$ structure

The 200 ns trajectory of the HDAC6 receptor was represented by 2000 frames. An RMSD plot of the conformations from frames 1-2000 fitted to frame 1 showed that during the first 20 ns, the protein was still equilibrating (see Fig. S1). We therefore performed PCA on frames 201–2000. Note that there is a more gradual increase in the RMSD from about 120 ns to the end of the simulation at 200 ns. This is possibly due to regions of the protein, such as loops, exhibiting slow relaxation. PCA was performed on all protein atoms and the zinc ion, but water molecules and ions, including the two crystallographic potassium ions, were not included. This amounted to 5478 atoms. This all-atom PCA was used for the linear response as described in previous publications [20, 28, 29] and applied to the HDAC6 trajectory. Figure 4 (A) shows the trajectory projected onto the first two principal modes which shows three distinct clusters. Equivalent figures by Shahab et al. [17] for 100 ns trajectories of HDAC6 complexes also indicated the presence of clusters although more than the three seen here. The first two principal modes (out of a total 16428) contribute 16% and 6%, respectively, to the total MSF. As described in a recent review [39] the conformations were projected onto the first and second modes (PC’s) to find the maximum and minimum extent of the modes. This produced two structures for each mode, $\:{\varvec{r}}_{1}^{min}$ and $\:{\varvec{r}}_{1}^{max}$ for mode 1, and $\:{\varvec{r}}_{2}^{min}$ and $\:{\varvec{r}}_{2}^{max}$ for mode 2. Although HDAC6 does not visually seem to comprise structural domains, these maximum and minimum structures were input into the DynDom program [41] at the DynDom webserver [42], which revealed a clear domain movement for mode 1 (PC1), but not for mode 2 (PC2). The resulting dynamic domains, hinge axis, and hinge bending regions are shown in Fig. 5. The domain boundary bisects the binding pocket and although the angle of rotation about the hinge axis is small (8.2°), the movement produces an appreciable widening and narrowing of the interdomain cleft at the active site as can be seen in Fig. 5. The movement in the positive direction of mode 1 causes the binding pocket to widen and in the negative direction it causes the pocket to narrow (see Fig. 4 (A)). Interdomain bending residues are: 444–448, 482–484, 551–552, 564–565, 615–618, 739–740, and 768–772 (note DockIT residue numbering is PDB residue numbering minus 441). The movement is 77% a twisting motion [43] controlled by the central β-sheet with the hinge axis on the surface of the sheet, oriented perpendicular to the strands, and passing close to the interdomain bending region on each strand. Like many proteins that undergo a small twisting of their domains, the movement is classified as predominantly shear [44] indicating that there is a relative sliding of the domains at their interface.

As already stated, to evaluate the response in real time we use the first *M* principal modes. Here we use *M* = 100, which accounted for 66% of the total MSF. Regarding the stability of this subspace, we divided the trajectory on which the PCA was performed into two equal halves and performed PCA on each. The root mean-square inner product (RMSIP) is a measure of the overlap of two subspaces and for identical subspaces (100% overlapping) achieves its maximum value of 1.0. For *M* = 100, the RMSIP value was 0.62.

In addition to the all-atom PCA used for linear response, we also performed a PCA on just backbone atoms (N, C_α_ and C) so that we could easily compare experimental structures in the subspace of the first two principal modes. In Supplementary Material Fig. S2 we present the crystallographic ligand-free, belinostat and HPOB bound structures projected on the first two principal modes of the backbone PCA. It shows that both the structures are closely located within the two clusters on the left in Fig. 4 (A) (which are somewhat merged in the backbone PCA). Thus, both experimental structures and the ligand-free structure are associated with the clusters on the left which have a narrower binding pocket than the cluster on the right.

The first and second principal modes (out of a total 3198) for this backbone PCA contribute 34% and 7% to the total MSF, respectively, indicating a very dominant first principal mode. A DynDom analysis for the movement between the minimum and maximum structures along mode 1 produced an almost identical result to the all-atom case. The RMSIP for *M* = 100 over the two halves of the backbone atom trajectory is 0.84, indicating a rather stable subspace.

To address the possible issue of whether a single MD simulation of 200 ns provides sufficient sampling, we performed two additional 200 ns simulations and evaluated the backbone RMSIPs between their 100-dimensional subspaces. The RMSIP values were 0.81 and 0.77, indicating a relatively stable subspace. This is in accordance with a previous study [29] where the docking-induced domain movement in maltodextrin binding protein and glutamine binding protein (MBP is the same size as HDAC6, GBP is slightly smaller) showed an excellent agreement with its respective experimentally determined domain movement even though the subspace for the linear response was derived from a shorter 100 ns trajectory.

### Initial placement of inhibitor molecules for automated docking

**Fig. 6:**
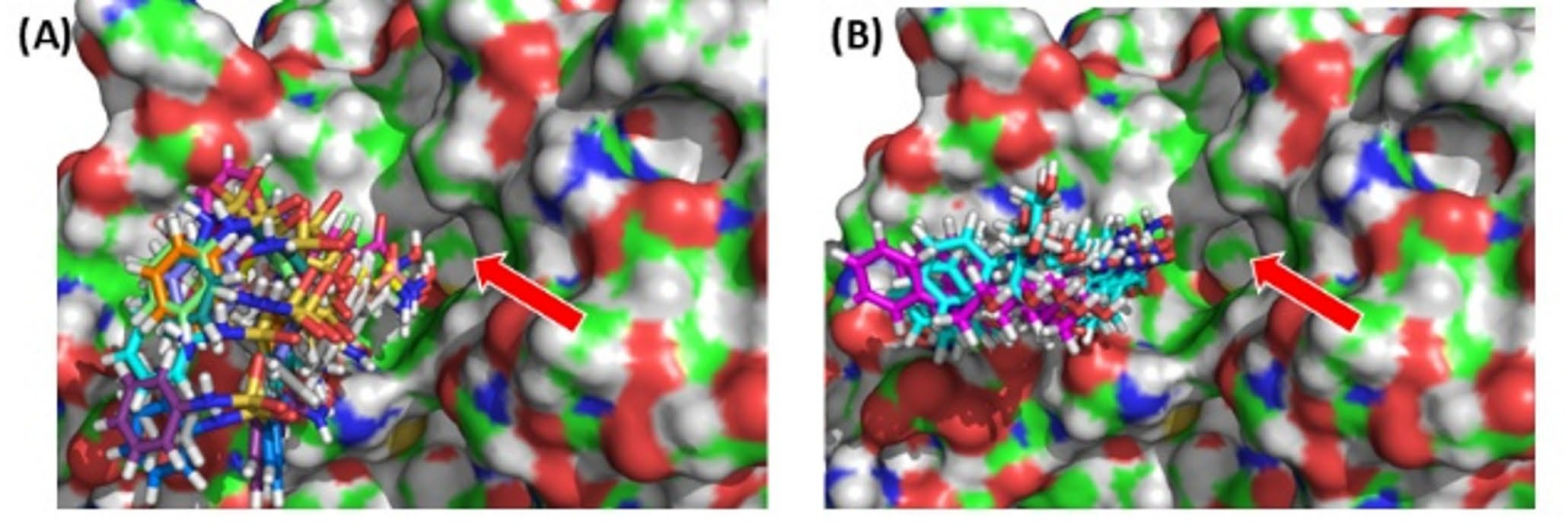
Shows the starting poses of inhibitors (stick depiction) that successfully entered the binding pocket of HDAC6 (molecular surface depiction). The red arrow indicates the binding pocket. (**A**) The 14 starting poses for belinostat (**B**) The 12 starting poses for HPOB. The 7 in cyan form a hydrogen bond to Tyr745, the hydroxamic acid group oriented as found in the crystallographic structure (PDB:5EF7). The 5 in magenta rotate almost 180° about the long axis of the hydroxamic acid group and form hydrogen bonds with Tyr745 and Asn530

For both inhibitors, the following procedure was carried out to find positions from which to start automated docking. The molecule was inserted into the binding pocket and then moved so that the hydroxamic acid was clearly outside of the pocket. If it moved back into the binding pocket when automated docking was engaged, then this position was used as a starting position from which a new position and orientation was trialed by implementing a small translation and rotation of the drug molecule. If from this new position the drug entered the binding pocket, this became the new starting position. This process was repeated 40–50 times for each molecule. Belinostat entered the binding cavity in 14 out of 46 trial positions, and HPOB entered the binding cavity in 12 out of 43 trials. Figure 6 shows the starting positions of both inhibitors for those starting positions that entered the cavity.

### Docking of belinostat

**Fig. 7:**
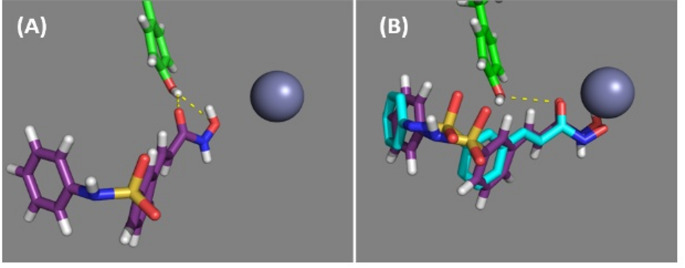
In purple stick depiction is the belinostat ligand. Green stick depiction is Tyr745. Hydrogen bonds are indicated by broken yellow lines and the zinc ion is depicted as a grey sphere (**A**) Belinostat in the intermediate pose after first phase of automated docking showing its hydrogen bonds with Tyr745 (**B**) Belinostat ligand has moved closer to the zinc ion in its final binding pose which is close to its crystallographic binding pose depicted in cyan stick

The 14 starting positions, shown in Fig. 6 (A), from which belinostat entered the binding pocket, had RMSDs with the ghost at the experimentally bound position in the range 8.7–16.2 Å. In Fig. 4 (B) the conformation of HDAC6 is projected onto the plane of the first two principal modes for selected positions of belinostat relative to HDAC6 during binding. It shows that HDAC6 changes conformation during the binding process. In all 14 cases, the final position of belinostat after automated docking resulted in the same binding pose with belinostat hydrogen bonded to the side chain hydroxyl group of Tyr745 as shown in Fig. 7 (A). During this stage Fig. 4 (B) indicates that domain twisting acts to narrow the binding pocket. Visually comparing this conformation with the relaxed conformation confirmed that a narrowing of the pocket had occurred and a DynDom analysis of the movement from the relaxed receptor conformation to this Tyr745 bound conformation showed an almost identical domain decomposition to that seen in Fig. 5, with the hinge axis pointing approximately in the opposite direction indicating narrowing of the binding pocket for this domain-twisting movement. For those starting positions further away from HDAC6, belinostat first forms a hydrogen bond with the side chain of Asn530, then forms a hydrogen bond with Asn645, before finally hydrogen bonding with Tyr745. Comparing the Tyr745 hydrogen bonded pose of belinostat with its ghost in the binding pocket reveals that it is at an intermediate binding site where it is partially inserted into the binding pocket (3.6 Å RMSD with the ghost). As this is at an energy minimum (total energy is -28 kcal/mol), there must be an energy barrier between the Tyr745 hydrogen-bonded pose and the crystallographic binding pose. Switching back to manual mode, belinostat was gently pushed further into the binding pocket causing an increase in the strain energy. In doing this, it forms a hydrogen bond with the main chain of Phe643, and the Leu712 moves away from the pocket mainly through the movement of the loop 709–716, on the tip of which Leu712 is located. After this manual intervention, where the RMSD with the ghost is 2.7 Å, another round of automated docking was implemented in which belinostat breaks its hydrogen bond with Phe643 and moves to its final binding pose that has a 1.0 Å RMSD with the ghost, i.e., very close to the experimentally determined binding pose (total energy is -40 kcal/mol), (see Fig. 7 (B)). During this stage the interaction and strain energies decrease. This relaxation results in a domain twisting that acts this time to widen the binding pocket (see Fig. 4 (B)). The estimated height of the energy barrier between the intermediate pose and the final binding pose is ∼ 10 kcal/mol.

It is to be noted that this final binding pose is identical to when belinostat is superimposed on its ghost and automated docking engaged until an energy minimum is found.

There are some observations that can be made about this process. For some paths to the intermediate binding site, there are simultaneous hydrogen bonds between Asn645 and the sulfonamide group of belinostat and between Tyr745 and the hydroxamic acid group of belinostat suggesting a relay of interactions occurs during binding. From the path shown in Fig. 4 (B) one can see that as belinostat moves into the binding pocket it is accompanied by domain twisting along the first principal mode of HDAC6 that acts to narrow the size of the pocket. Interestingly, the conformation of HDAC6 moves outside the distribution of conformations seen for the ligand-free protein, indicating an induced-fit mechanism. In these conformations the HDAC6 closes further upon belinostat with the binding pocket adjusting size so that it snuggly fits to belinostat as judged by their complementary molecular surfaces. The docking trajectories for the conformation of HDAC6 do not approach conformations in the cluster on the right in Fig. 4 (B), and like the experimental structures (see Fig. S2), the final docked structure of HDAC6 is very close to the relaxed, ligand-free receptor conformation (RMSD = 0.09 Å, calculated on C_α_ atoms for comparison to the experimental values given in Table I).

### Docking of HPOB

The 12 starting positions, shown in Fig. 6 (B), from which HPOB entered the binding pocket, had RMSDs with the HPOB ghost at the crystallographic bound position in the range 9.7–14.2 Å. In Fig. 4 (C) and (D) the conformation of HDAC6 during the binding process is projected onto the plane of the first two principal modes for selected poses of HPOB relative to HDAC6. As for belinostat, after automated docking, HPOB moves to an intermediate binding pose at an energy minimum where a hydrogen bond with the side chain hydroxyl group of Tyr745 results. The movement to this intermediate site is, as with belinostat, also accompanied by a domain twisting that acts to narrow the size of the binding pocket. Interestingly the 12 intermediate binding poses can be divided into two distinct groups (see Fig. 6 (B)), 7 with the hydroxamic acid group oriented as found in the crystallographic HPOB-HDAC6 complex structure (total energy is -25 kcal/mol) and 5 with the hydroxamic acid group, oriented in as in belinostat (total energy is -30 kcal/mol). HPOB in the former group will be referred to as being in the “crystallographic orientation” and the latter group will be referred to as being in the “belinostat orientation.” In this intermediate binding pose the hydroxamic acid in the crystallographic orientation is an almost exact 180° flip of its orientation in the belinostat orientation.

#### HPOB that binds in the crystallographic orientation

**Fig. 8:**
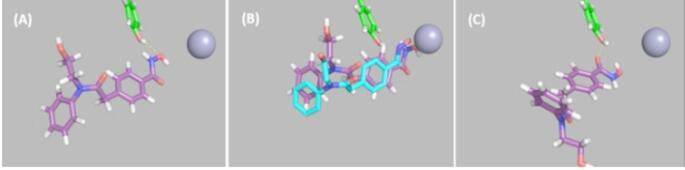
In purple stick depiction is the HPOB ligand. Green stick depiction is Tyr745. Hydrogen bonds are indicated by broken yellow lines and the zinc ion is depicted as a grey sphere (**A**) HPOB with its hydroxamic acid in the crystallographic orientation in the intermediate site having formed a hydrogen bond with Tyr745 (**B**) Final docked binding pose of HPOB with hydroxamic acid in the crystallographic orientation compared with the crystallographic pose of HPOB (cyan stick) (**C**) HPOB in with its hydroxamic acid in the same orientation as belinostat in the intermediate site with a hydrogen bond to Tyr745. It also formed a hydrogen bond with Asn530 (not shown). Note the orientation of HPOB in (A) and (B) is approximately a 180° flip about the long axis of the hydroxamic acid group in comparison to its orientation in (C)

At the intermediate pose with the hydrogen bond between HPOB and Tyr745, HPOB had a 2.4 Å RMSD with the HPOB ghost (see Fig. 8 (A)). As for belinostat we gave HPOB a gentle push further into the pocket from this intermediate pose. After this manual intervention the RMSD of HPOB with ghost HPOB was 1.6 Å. After re-engaging automated docking, they all moved to deeper into the pocket to reach a final binding pose that had a 1.4 Å RMSD with the ghost, i.e., very close to the crystallographic binding pose (the total energy is -35 kcal/mol) (see Fig. 8 (B)). As for belinostat, during this stage domain twisting acts to widen the binding pocket (see Fig. 4 (C)). The estimated height of the energy barrier between the intermediate pose and the final binding pose is ∼ 2–3 kcal/mol.

This final binding pose is identical to when HPOB is superimposed on its ghost and automated docking engaged until an energy minimum is found.

#### HPOB that binds in belinostat orientation

At the intermediate pose, HPOB in the belinostat orientation had a 5.0 Å RMSD with ghost HPOB (Fig. 8 (C)). In addition to the hydrogen bond with Tyr745 it had a hydrogen bond with the side chain of Asn530. Pushing HPOB further into the pocket and re-engaging automated docking, resulted in it moving back out of the pocket, indicating a very high energy barrier. The likely reason for HPOB not being able to fully enter the binding pocket in this orientation is that steric clashes between the cap group and the aperture of the HDAC6 binding pocket prevent it.

#### Role of cap group of HPOB in determining orientation

**Fig. 9:**
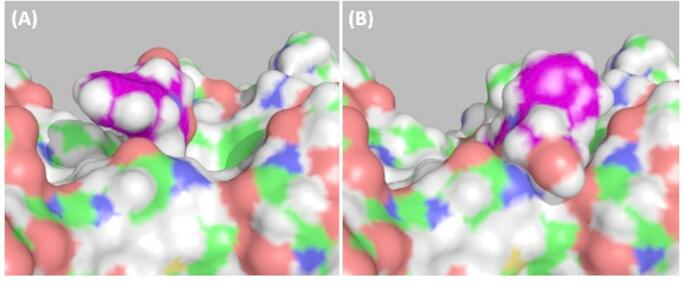
Molecular surface depictions of HDAC6 (green) and HPOB (purple) at the intermediate binding site. (**A**) In crystallographic orientation from which it is able to move deeper into the pocket (**B**) In belinostat orientation where it is unable to move deeper into the pocket due to the greater number of contacts between the cap and HDAC6

If we define a “contact” to be when any pair of heavy atoms are within 4 Å of each other, and the cap of HPOB to comprise atoms O3, O4, N2, and C6-C15 (atom names from PDB file: 5EF7), then with HPOB at the intermediate binding site, there are 9 contacts between the cap of HPOB and HDAC6 in the belinostat orientation, compared to only 4 for the crystallographic orientation. In the crystallographic orientation there is space between the cap and HDAC6 allowing HPOB to move deeper into the binding pocket (see Fig. 9 (A)). The contacts for HPOB in the belinostat orientation that appear to block further penetration deeper into the pocket are primarily between the O-N-C_2_-OH group of HPOB and Asn530 of HDAC6 (see Fig. 9 (B)). From these results it is tempting to draw the conclusion that HPOB binds with its hydroxamic acid in a flipped orientation compared to belinostat because there is a significantly lower energy barrier between the intermediate pose and the fully inserted pose due to there being fewer intermolecular contacts between the HPOB cap and HDAC6 in this orientation.

## Discussion

### Automated docking in DockIT

DockIT is primarily a tool for interactive molecular docking but here we have introduced a feature for automated docking within the DockIT environment. The automated docking implemented is one where flexibility in the receptor, both global and local, is modelled using the method of linear response based upon a 200 ns MD simulation of the protein in explicit solvent. We are currently limited to using a rigid ligand model. The automated docking method we use is a quasi-static process in that for each position of the ligand, the receptor reaches static equilibrium. For each conformation of the receptor the ligand undergoes a steepest-descent translational and rotational step according to the forces acting on it from the receptor. A number of other studies have attempted to model receptor flexibility in docking [45–47] but most of these methods are not appropriate for an interactive docking application. Our method is similar to the method of May and Zacharias [48] which also used energy minimization in a protein-protein interaction docking scenario (not interactive) using normal modes derived from an Elastic Network Model for one of the binding partners. They reported that modeling global flexibility improved agreement to the experimentally derived complex.

### HDAC6 flexibility

A DynDom analysis of the movement along the first principal mode shows clearly that HDAC6 has a domain movement that is controlled by the twisting of the β-sheet that spans the whole molecule. Interestingly the domain boundary bisects the binding pocket which means the domain movement changes its size and shape. As the domain movement seems to be a natural consequence of its structure, it is reasonable to propose that it may be engaged when HDAC6 binds its natural substrates. This was unexpected as the binding of inhibitors does not change the global conformation of HDAC6 as indicated by the small RMSD between the ligand-free and ligand-bound structures (see Table I). However, HDAC substrates are large proteins, and their binding will necessarily involve other regions of HDAC6 interacting with residues outside the enzyme active site. It is likely therefore that this domain movement occurs when other regions on HDAC6 interact with its substrate and that it plays a significant role in the overall reaction process. It is probably due to the relatively small size of the inhibitors, mimicking little more than the side chain of acetyllysine in the substrate, that the crystallographic drug-bound structures of HDAC6 do not show a domain movement relative to the ligand-free HDAC6.

### Receptor flexibility during docking

For the docking experiments carried out here the final bound conformations also do not show a domain movement relative to the relaxed conformation. This can be seen from the positions of the final docked conformation in the projections on the first two principal modes of HDAC6, which lie close to the relaxed conformation along the first principal mode coordinate. However, in both cases, during the docking process the conformation of HDAC6 moves towards more negative values of the first principal mode to conformations not sampled during the MD simulation. These conformations occur as the molecules enter the binding pocket which continuously adjusts its size and shape so that its molecular surface is complementary to that of the inhibitor’s. This is an induced fit mechanism that occurs during the binding process. The extent to which this provides a true insight into what occurs during binding cannot be established from this study.

### Effect of ignoring ligand flexibility

Currently DockIT is not able to model ligand flexibility. Instead, we have rigidly docked the ligands taking their conformation from the crystallographic structure of the complex. Although there are rotatable bonds within both the inhibitor molecules, when the ’cap’ enters the binding pocket, rotation about bonds cannot occur to any great degree without steric clash with HDAC6. Undoubtedly, rotation about these bonds will affect any energy barriers caused by steric interaction as the cap enters the pocket. Rotations that affect the relative orientation of the hydroxamic acid warhead and the cap are likely to have a greater effect.

### Solvent effects

There are solvent effects that cannot currently be handled within DockIT. Although the MD simulation was performed using an explicit solvent model and consequently solvent effects on conformational flexibility are included implicitly in the linear response, an explicit solvent model cannot be used within an interactive docking session for obvious reasons. To account for the screening effect of the water solvent on electrostatic interactions, we use a distance-dependent relative permittivity [28, 49]. However, hydrophobic interactions are not currently modelled in DockIT and consequently their contribution to the binding free energy is missing. It is not known the degree to which this will affect results.

### Relay of hydrogen bonds and interplay between warhead and cap group interactions

As belinostat’s cap enters the binding pocket there are many ways in which it can be oriented. Here, results suggest that the cap helps to orient it correctly through formation of a hydrogen bond between the side chain of Asn645 and the oxygen of the sulfonamide cap. Whilst this hydrogen bond continues, the hydrogen bond between the side chain of Tyr745 and the hydroxamic acid forms before the hydrogen bond with Asn645 eventually breaks and the energy minimum with the Tyr745 hydrogen bond intact is reached. This relay of hydrogen bonds is also seen in HPOB and suggests that there is interplay between cap and warhead interactions, with those of the cap helping to correctly orient the warhead. A relay of interactions has been seen before in a binding process, namely in NAD+- induced domain closure in the enzyme horse liver alcohol dehydrogenase [50].

### Tyr745 hydrogen bonded pose as intermediate

In all cases automated docking from starting poses where the ligand entered the binding pocket resulted in a final pose at an energy minimum where there was a hydrogen bond between Tyr745 side chain and the hydroxamic acid of the drug molecule. In this pose, the ligand is not as deeply buried in the pocket as the respective ghost molecule. This led us to think that this is an intermediate pose and that there could be a lower energy pose, with the inhibitor buried deeper in the pocket, filling in the remaining space and closing the gap between the hydroxamic acid and the zinc ion. Gently pushing the molecule deeper into the pocket from the Tyr745 hydrogen-bonded pose and re-engaging automated docking resulted in it moving to a final pose where the hydroxamic acid group is very close to that of the ghost, i.e., as in the crystallographic structure. For both belinostat and HPOB the final, fully inserted pose had a lower total energy than the Tyr745 hydrogen-bonded pose. Thus, in our study, the Tyr745 hydrogen-bonded pose is an intermediate binding pose that we believe exists in substrate recognition by HDAC6. The energy barrier between the intermediate and final pose varies depending on the inhibitor and seems to be dependent on the cap’s interaction with the HDAC6 surface. The energy barrier comprises mainly of strain energy indicating that deformation of HDAC6 is required for the inhibitor to enter deeper into the pocket. After overcoming the energy barrier, the strain energy reduces, the binding pocket widens through domain twisting, and the inhibitor is able to move through automated docking to its final binding pose deep inside the pocket where HDAC6 is in a relaxed conformation. This is supported by the experimental evidence which shows very little difference in conformation between the ligand-bound and ligand-free structures of HDAC6.

The results on HPOB suggest that it binds with its hydroxamic acid in the flipped orientation because there are fewer interactions between the cap of HPOB and regions on the surface of HDAC6 surrounding entrance to the pocket. In the orientation with the hydroxamic acid group oriented as in belinostat, the cap has more interactions with the residues on the surface of HDAC6, in particular Asn530, hindering it from moving deeper into the pocket.

We believe the Tyr745 hydrogen bonding pose gives important clues to HDAC substrate recognition. This tyrosine residue is conserved in all HDAC enzymes with high catalytic turnover and is substituted by histidine in HDAC4,5,7 and 9 which have very low catalytic activity [51]. Furthermore the zebrafish CD2 HDAC6 Tyr745Phe mutant leads to a loss of activity against a substrate derived from the α-tubulin Lys40 acetylation site [10]. Enzymes with high catalytic activity presumably track across a protein surface making contacts with side chains in a reversible manner. Among canonical protein side chains as well as PTMs, acyllysine residues are unique in being able to occupy the substrate binding pocket and engage in hydrogen bonding between the amide and the Tyr gatekeeper residue. This leads to an energy minimum, in the same way as observed with the hydroxamic acids in our inhibitors. Further domain movements then serve to widen the active site (see Fig. 4 (B) and (C)), allowing acyllysine substrates to enter more deeply and coordinate to the zinc cation, resulting in catalysis.

Overall, our results suggest that initial recognition of substrates or inhibitors by HDACs occurs through relatively low affinity interactions with amino acid residues at the enzyme surface. These can vary between substrates and inhibitors, as shown by our contrasting results with belinostat and HPOB. The substrate/inhibitor enters the pocket and hydrogen bonds with the gatekeeper Tyr/His residue (Tyr745 in HDAC6). From this intermediate state, the substrate/inhibitor then penetrates more deeply to approach the catalytic zinc cation in the active site. Inhibitors with increased surface recognition would be one approach to discriminate between isozymes and achieve selectivity.

## Electronic supplementary material

Below is the link to the electronic supplementary material.

Supplementary Material 1

Supplementary Material 2

## Data Availability

DockIT is free for non-commercial use and can be downloaded from https://expresslicensing.uea.ac.uk/product/dockit. Structure files, topology files, eigenvalue and eigenvector files to run DockIT with HDAC6 as the receptor and belinostat and HPOB as the ligand are available from this link: 10.5281/zenodo.15528370. Also available are ghost ligand structure files and DockIT workspace files. The results described in this article can be reproduced by loading these workspace files into DockIT and starting automated docking by pressing the “Auto” button.
