# Supplementary material for: Protein domain movement involved in binding of belinostat and HPOB as inhibitors of histone deacetylase 6 (HDAC6): a hybrid automated-interactive docking study
Source: J Comput Aided Mol Des. 2025 Jul 15;39(1):52. doi: 10.1007/s10822-025-00636-x (PMC12263812; doi:10.1007/s10822-025-00636-x)
Supplement: Supplementary file 1 — Supplementary Material 1 [file 10822_2025_636_MOESM1_ESM.pdf]

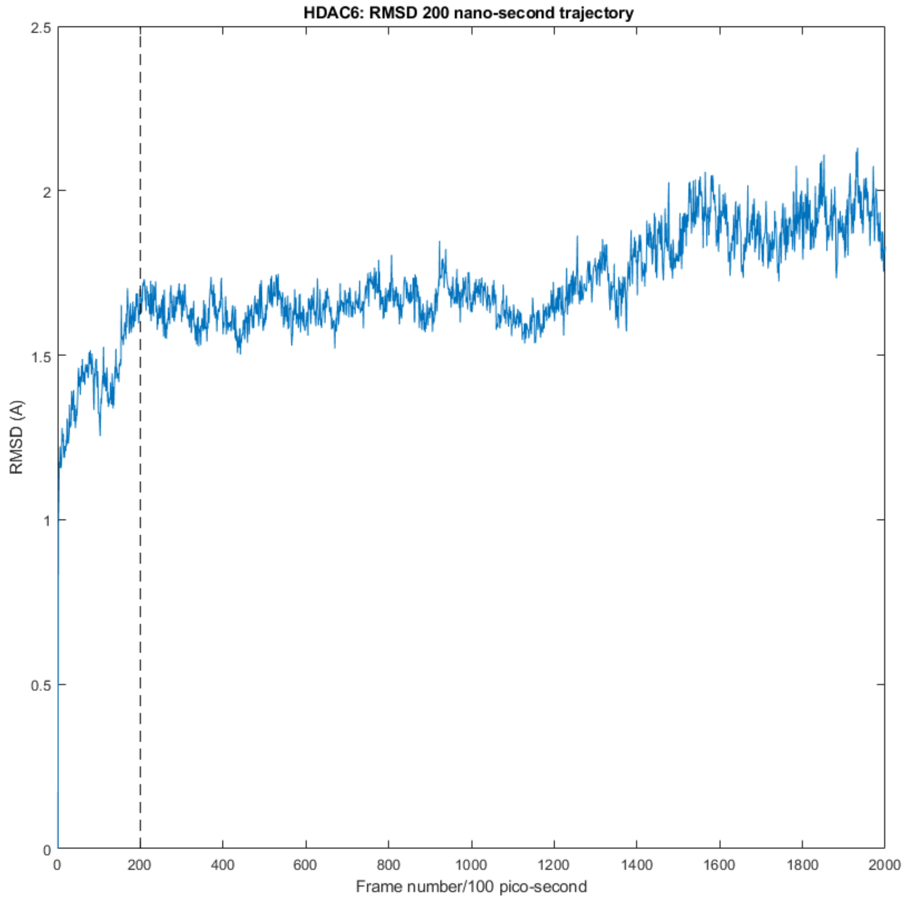

Figure S1 RMSD plot of the conformations from frames 1-2000 fitted to frame 1 showing that during the first 20 ns, the protein is still equilibrating.
