# Supplementary material for: Protein domain movement involved in binding of belinostat and HPOB as inhibitors of histone deacetylase 6 (HDAC6): a hybrid automated-interactive docking study
Source: J Comput Aided Mol Des. 2025 Jul 15;39(1):52. doi: 10.1007/s10822-025-00636-x (PMC12263812; doi:10.1007/s10822-025-00636-x)
Supplement: Supplementary file 2 — Supplementary Material 2 [file 10822_2025_636_MOESM2_ESM.pdf]

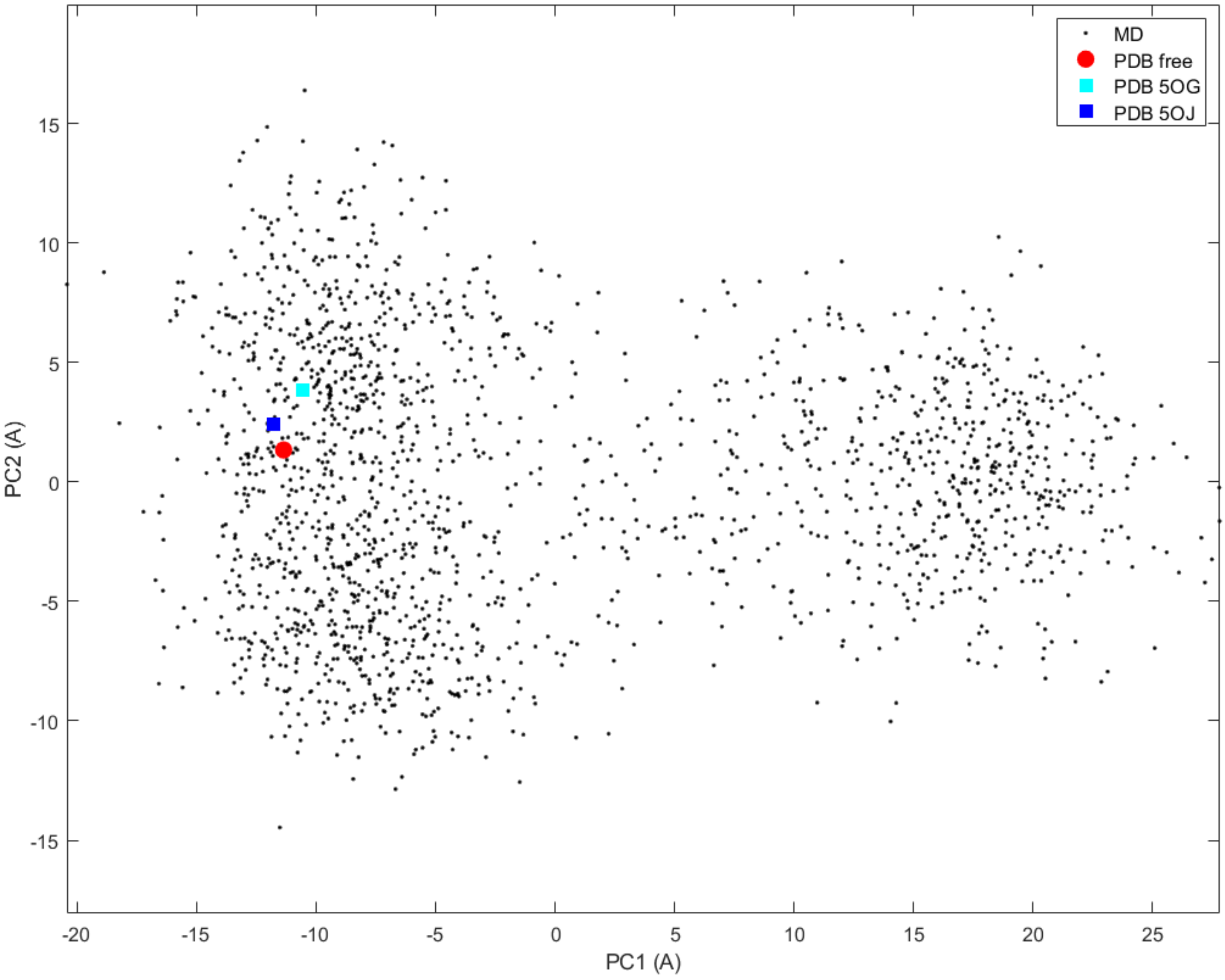

Figure S2 1800 frames from 20.1 ns-200 ns portion of trajectory projected on to the plane defined by the first two principal modes from a principal component analysis of the trajectory using just backbone atoms (N, C_{α} and C). The experimental structures (chain A, results using chain B are almost identical) are projected onto this plane. 5OG is belinostat and 5OJ is HPOB.
